# Expectations and Experiences With Online Education During the COVID-19 Pandemic in University Students

**DOI:** 10.3389/fpsyg.2021.815564

**Published:** 2022-01-05

**Authors:** Karla Lobos, Rubia Cobo-Rendón, Javier Mella-Norambuena, Alejandra Maldonado-Trapp, Carolyn Fernández Branada, Carola Bruna Jofré

**Affiliations:** ^1^Laboratorio de Investigación e Innovación educativa Dirección de Docencia, Universidad de Concepción, Concepción, Chile; ^2^Programa de Doctorado Educación en Consorcio, Universidad de Católica de la Santísima Concepción, Concepción, Chile; ^3^Departamento de Física, Facultad Ciencias Físicas y Matemáticas, Universidad de Concepción, Concepción, Chile; ^4^Departamento Currículum e Instrucción, Facultad de Educación, Universidad de Concepción, Concepción, Chile; ^5^Departamento de Bioquímica y Biología Molecular, Facultad de Ciencias Biológicas, Universidad de Concepción, Concepción, Chile

**Keywords:** COVID-19, higher education, university student, online teaching and learning, student self-efficacy

## Abstract

Due to COVID-19, university students continued their academic training remotely. To assess the effects of emergency remote teaching (ERT), we evaluated the expectations and, subsequently, the experiences of university students about online education. This study employed a simple prospective design as its method. We assessed the expectations of 1,904 students from different discipline areas (1,106 women and 798 men; age *M* = 21.56; *SD* = 3.07) during the beginning of the first semester, March 2020 (T1), and their experiences at the end of the same academic period, September 2020 (T2). We used convenience non-probability sampling. Participants responded to the questionnaire on Expectations toward virtual education in higher education for students and the questionnaire on virtual education experiences in higher education. The results showed that students’ responses reflected low expectations regarding peer relationships and comparison with face-to-face education (T1). This perception was maintained during the evaluation of experiences (T2). Students reported positive experiences regarding online teaching and learning, online assessment, and their self-efficacy beliefs at T2. Statistically significant differences between measurements were found, with the expertise presenting higher averages than expectations. Furthermore, differences by gender were identified, reporting a positive change in the scores of women. In addition, results reflected differences according to the disciplinary area, showing Social Sciences and Medical and Health Sciences students a more significant size effect. Findings regarding the empirical evidence and the implications for future teaching scenarios in Higher Education are discussed.

## Introduction

Higher education institutions had to face the challenge of providing continuity to the educational process remotely due to the COVID-19 pandemic. This scenario implied a drastic transformation without the possibility of preparation, having both teachers and students quickly develop online education competencies (Hattar et al., 2021). Emergency remote teaching (ERT) is the name given to this instructional response (Bustamante, 2020; Hodges et al., 2020). ERT applies to any unexpected and urgent transition to online instruction due to a disaster. Given its nature, one of the characteristics of ERT is the lack of time and skills of instructors to adequately prepare and implement their course syllabus in a virtual format (Hodges et al., 2020). Thus, ERT differs significantly from online teaching, in which the focus is on delivering a quality learning experience following a predefined instructional design (Miramontes Arteaga et al., 2019).

Currently, online courses are created using an instructional design, such as ADDIE, and implemented through Learning Management Systems (LMS), like Canvas. In these courses, designers and teachers apply technological and pedagogical innovations to obtain high-quality standards. In this teaching modality, educational experiences occur synchronously and asynchronously using multiple devices to access the internet. Therefore, students can interact with teachers, content, and peers from wherever they are (Singh and Thurman, 2019). It requires stable digital infrastructure and platforms. Thus, its implementation demands many resources and a carefully designed plan to deliver a quality experience (Mousa et al., 2020). As necessary and valuable as ERT is, its design does not necessarily consider the critical elements of quality online education (Hodges et al., 2020). Despite the advances in online education in many higher education institutions worldwide, universities, in general, were not prepared for the necessary, mandatory, and abrupt change at the onset of the COVID-19 pandemic (Maier et al., 2020).

Quality online teaching considers evaluating course characteristics, including the design of learning materials, the virtual environment, and the alignment of curricular components with learning outcomes. It also considers aspects related to the interaction experience of students with their peers and teachers (Rodrigues et al., 2019).

### Literature Review

Due to the COVID-19 pandemic, students’ expectations about how their academic year would unfold were rapidly modified and adjusted. This is relevant due to empirical evidence that supports that student expectations are predictors of academic success (Paechter et al., 2010; Alhabeeb and Rowley, 2018; Wei and Chou, 2020). Student expectations can be defined as the beliefs that students hold about successfully coping with academic responsibilities. From the perspective of the expectancy-value theory (Wigfield and Eccles, 2000), students have beliefs about their ability and success in meeting academic demands. These beliefs can be impacted by the subjective perception of the value of the academic activity to the student (Valle et al., 2015). The expectancy-value theory is widely used to understand how psychological and contextual factors enhance student engagement and learning outcomes (Chiu et al., 2021). Furthermore, expectations also impact student attitudes about the ways of learning (Fernández Jiménez et al., 2017). It has been reported that students’ perceptions regarding online learning modalities are related to their learning success (Nur Agung et al., 2020). Therefore, expectations and experiences of university students regarding online learning courses during the pandemic could translate into opportunities or obstacles in the sense of moving closer or further away from a practical online education experience in the future (Rodrigues et al., 2019; Pham and Ho, 2020).

Several studies have reported a variety of results regarding the expectations and subsequent experience of university students. For example, descriptive research conducted with 1612 undergraduates from 59 on-site Spanish universities says that students consider that the institutions did not adapt adequately to the ERT scenario (84%), especially regarding teaching methods and the implementation of assessments (64.5%). Furthermore, they state that the adopted institutional measures were not sufficient, affecting their academic performance (88.5%) during this period. In terms of experience, in the same research, students were not satisfied with virtual education, especially regarding courses’ assessment (Villa et al., 2020). These results relate to another study that reported that students would not repeat this experience due to the absence of interaction with teachers, excess of tasks, and the accelerated pace for learning (Imsa-ard, 2020; Suárez et al., 2021).

Consistent with the above, another study indicates that students perceived an overload in their academic responsibilities due to excessive activities and assignments, which made the process more exhausting (Rahiem, 2020). Moreover, another research from the pandemic experience indicates that young people reported a low perception of quality and quantity in their learning during ERT regarding the strategies implemented by their universities, which did not meet their expectations (31.3%) (Almomani et al., 2021). Additionally, researchers found that, unlike men, women perceived greater satisfaction with the strategies implemented by universities (66%), were more committed to delivering their assignments (70.6%), and were more optimistic about the assessment process implemented by teachers in their courses (70.2%) (Almomani et al., 2021). Another research concludes that online teaching during the COVID-19 pandemic was only possible when online learning had a robust digital infrastructure and a learning system designed for that purpose; otherwise, it was an attempt to replicate face-to-face teaching in the virtual environment (Abdulrahim and Mabrouk, 2020).

Despite the emergency scenario caused by the pandemic, not all studies reported negative experiences (Abdulrahim and Mabrouk, 2020; Sepulveda-Escobar and Morrison, 2020). During ERT training, students from various institutions worldwide (*N* = 30,383) claimed to be satisfied with the support provided by their instructors and institutions. In this case, specific sociodemographic characteristics such as gender, academic area, and other elements of the students favorably impacted these beliefs (Aristovnik et al., 2020). Students positively assessed the actions implemented by the universities’ Information and Communication Technologies Departments (Shehzadi et al., 2020). In addition, they thoroughly evaluated the online platforms used since they allowed them to perform their tasks efficiently and quickly, having fun while studying (Maier et al., 2020). It is important to note that some authors report differences in experiences according to the scientific disciplines to which students belong (Vladova et al., 2021a).

Regarding social aspects, it seems that students were not satisfied with the preparation of teachers during the ERT modality due to difficulties in the interaction with their teachers and peers (Alqurshi, 2020; Hamdan et al., 2021). This aspect is consistent with other research highlighting the importance of interaction between instructors and students in the online education experience (Sun, 2016; Bao, 2020).

Due to ERT, a negative effect on students’ self-efficacy beliefs about online education has been reported at the individual level (Aldhahi et al., 2021), while others found no changes (Talsma et al., 2021). Self-efficacy is a relevant element regarding students’ academic satisfaction and performance (Cervantes Arreola et al., 2018; Hamdan et al., 2021). When students believe in successfully facing the challenges of online education, they display a series of mechanisms to favor a more efficient and effective coping of their learning process. Consequently, beliefs conversion during the ERT may play an essential role in post-pandemic online learning.

In the context of the COVID-19, the academic, social, and individual experiences during ERT affect the perception of online education, which could impact the implementation of this modality in Higher Education in the future.

### The Present Study

The empirical evidence described highlights the importance of assessing students’ experience during the ERT, especially the quality of the learning experience, the integration of teaching approaches, the design, the application of assessment tools, and how the relationship between students and their teachers is fostered (Sun, 2016; Alqurshi, 2020; Aristovnik et al., 2020; Bao, 2020; Rahiem, 2020; Van Heuvelen et al., 2020; Villa et al., 2020; Almomani et al., 2021; Suárez et al., 2021). These aspects will provide vital information for the design and implementation of effective online learning processes that respond to the needs of students and universities in this context in the future.

This study focuses on the importance of learning about students’ expectations and experiences during the implementation of the ERT for the COVID-19 pandemic. Specifically, we inquire on how students’ expectations and experiences can affect their academic, social, and personal aspects to provide evidence to support actions for the transition to face-to-face and blended learning. In this context, this research aims to analyze the expectations and experiences of students in a traditional university in the south of Chile at a general level and in consideration of the participants’ gender and disciplinary area.

Based on the above and the heterogeneity of students’ experiences reported in the literature, we describe the following hypotheses:

H_1_. There will be changes in the experiences to the expectations of university students during ERT due to the COVID-19 pandemic.

H_2_. Differences will be found between men and women regarding university students’ expectations and experience scores during the ERT due to the COVID-19 pandemic.

H_3_. Differences in university students’ expectations and experience scores will be observed according to disciplinary areas during ERT due to the COVID-19 pandemic.

## Materials and Methods

A simple *ex post facto* longitudinal quantitative research design was used. Researchers find it impossible to manipulate the independent variable in *ex post* facto studies, describing the associations between variables. It is simply longitudinal since two measurements were performed, starting by measuring the expectation (March 2020; T1) and then the experience (September 2020; T2) of the students with online education during the ERT, to subsequently study the relationships found between the variables (Montero and León, 2007).

### Participants

A total of 1,904 students belonging to a traditional Chilean university participated, of which 1106 (58.1%) were women, and 798 (41.9%) were men, with mean age *M* = 21.56 (*SD* = 3.07). On the other hand, 635 (33.35%) of the participants were in their first academic year. According to their undergraduate program, students’ classification according to the areas of the Organization for Economic Co-operation and Development (OECD) is presented in Table 1.

**TABLE 1:** Distribution of students by gender and disciplinary area.

OECD area	Men	Women
Agricultural sciences	50	91
Medical and health sciences	126	290
Natural sciences	154	158
Social sciences	147	362
Humanities	10	51
Engineering and technology	310	155

Totals by gender	797	1107
Total, sample	1904	

### Instruments

#### Expectations Toward Virtual Education

The Expectations toward Virtual Education in Higher Education for Students (CEEVESE) questionnaire aims to know higher education students’ expectations about virtual education during ERT. It consists of 28 items distributed in six dimensions about virtual education. The items were elaborated based on available scientific literature and evaluated employing expert judgment (Lobos et al., 2022). Table 2 describes the dimensions that constitute the scale.

**TABLE 2:** Description of the dimensions of the CEEVESE questionnaire.

Dimension (number of items)	Description
Peer relationship (6 items)	Expectations assessment about the student’s ability to interact with peers online.
Online learning (5 items)	Expectations assessment regarding learning support provided by online resources.
Online teaching (8 items)	Evaluation of students’ expectations about the university’s commitment and teachers’ abilities such as their delivery of courses as planned, attention to the learning process, and ability to use the virtual classroom tools.
Self-efficacy for online learning (5 items)	Assessment of students’ beliefs about their perception to meet the challenges of online education.
Online assessment (2 ítems)	Evaluation of expectations on the design, planning, and implementation of online testing.
Comparison/Contrast with face-to-face education (2 items)	Evaluation on the expectations about student’s performance and learning in online education compared to traditional or face-to-face education.

A Likert scale with five response options (1 = Strongly disagree to 5 = Strongly agree) was employed. The average of each dimension and the full scale was analyzed, in which scores higher than 3 indicate positive expectations. Previous studies have examined the factorial structure of the scale, finding an adequate adjustment of the 6 factors [X^2^(335) = 5354.88, *p* < 0.001, CFI:0.961; TLI:0.956; SRMR:0.041; RMSEA:0.06]. The reliability analysis of the responses was: peer relationship α = 0.894, online learning α = 0.922; online teaching α = 0.907; self-efficacy for online learning α = 0.882, online assessment α = 0.787; comparison with face-to-face education α = 0.779; full scale: α = 0.954 (Lobos et al., 2022).

#### Experience in Virtual Education

The Virtual Education Experiences in Higher Education for Students (EEEL) questionnaire adapts the CEEVESE (Lobos et al., under review^1^). Its purpose is to learn about the experiences of higher education students with virtual education during ERT. It consists of the same 28 items of the CEEVESE but presented in the past tense, using again a Likert scale of 5 response options (1 = Strongly disagree to 5 = Strongly agree). For their interpretation, the averages of each dimension and the full scale were analyzed. In both cases, the presence of scores above 3 points reflects a positive student experience. The items’ distribution corresponds with the six original dimensions.

The factorial structure of this version confirmed an adequate adjustment of the 6 factors [*X*^2^(333) = 3599.92, *p* < 0.001, CFI: 0.966; TLI: 0.961; SRMR: 0.036; RMSEA: 0.059]. Reliability analysis of the responses by dimensions was as follows: peer relationship α = 0.869, online learning α = 0.883; online teaching α = 0.876; self-efficacy for online learning α = 0.872, online assessment α = 0.753; comparison with face-to-face education α = 0.671; full scale: α = 0.931 (Lobos et al., under review, see text footnote 1).

### Procedure

This research was endorsed by the Ethics Committee of the participating university, corroborating the ethical criteria for research with human beings. The expectations and experience instruments were applied in digital format and sent to the students’ institutional emails on a single occasion. For the two measurement moments (T1 and T2), the questionnaires were available for 3 weeks at the beginning of March 2020 and at the end of September 2020. Students responded after reading and signing an informed consent form. A convenience non-probability sampling was used. The participants were students who were enrolled in a course during the first semester of 2020. To track the students, the enrollment number and e-mail address of each participant were compared. Only students presenting both measurements were included.

### Analysis Plan

We performed a descriptive analysis of the variables. Verification of the assumption of normality for the dimensions and total scales in both measurements (T1 and T2) was made using the Kolmogorov-Smirnov test with the Lilliefors modification (Thode, 2002). Analyzed data did not have a normal distribution (*p* < 0.001). Despite this, the Student’s *t*-test for paired samples was performed to evaluate the differences in the T1 and T2 scores due to the sample size.

The assumptions were verified using the mixed ANOVA tests to assess the effects between groups on gender and OECD areas versus the intra-group effect (changes between expectations and experience). No extreme outliers were found. Levene’s test was analyzed, finding no significant result (*p* > 0.05). The homogeneity of covariance of the between-subjects factor (gender-OECD area) using Box’s *M* test was also evaluated, with a not statistically significant result (*p* > 0.001). Therefore, no violation of the homogeneity of covariances assumption is assumed. Verification of the sphericity assumption was automatic since the Greenhouse-Geisser sphericity correction was applied to violating assumption factors during the ANOVA test calculation.

The size effect was analyzed considering the cutoffs by Cárdenas Castro and Arancibia Martini (2014), in which scores >0.14 are considered large, 0.06 medium, and 0.01 small. The data analysis was performed with R Studio software version 4.0.3 (2020-10-10) (R Core Team, 2020).

## Results

The present research aims to analyze the students’ expectations and experiences, considering the gender and disciplinary area of the participants. We presented the results in the context of the research hypotheses described in section “The Present Study.”

### Differences Between University Students’ Expectations and Experiences During Emergency Remote Teaching During the COVID-19 Pandemic

Hypotheses H_1_ sought to answer the existence of changes between the expectations and experiences of university students produced by ERT during the COVID-19 pandemic. In the first measurement (T1), the general students’ expectations presented an average below 3 points, identifying them as low (*M* = 2.92, *SD* = 0.65). The dimension that presented the highest score was self-efficacy for online education (*M* = 3.42; *SD* = 0.84), whereas the dimensions that showed the lowest scores were peer relationship (*M* = 2.1; *SD* = 0.83) and comparison with face-to-face teaching (*M* = 1.91; *SD* = 1.07).

Regarding the measurement of the students’ experiences with the ERT after the academic semester (T2), the perception was positive since the score was higher than 3 points (*M* = 3.18, *SD* = 0.66). Furthermore, the analysis by dimensions, identify that dimensions’ averages of the experiences (T2) were higher than its corresponding dimensions of the questionnaire of expectations (T1). However, despite having improved, the dimensions of peer relationship (*M* = 2.26, *SD* = 0.95) and comparison with face-to-face education (*M* = 2.71, *SD* = 1.24) remain as negative perceptions, since scores were still lower than 3. Table 3 shows dimensions’ averages and deviations of the scales applied and the assessment of the differences between them.

**TABLE 3:** Descriptive and inferential statistics on students’ expectations and experiences during the ERT.

Variable	Expectation (T1)		Experience (T2)		*T*-test	
	*M*	*SD*	*M*	*SD*	*T*	*d*
Online learning	3.14	0.87	3.43	0.83	15.0 (1903) ***	0.34
Comparison with face-to-face education	1.91	1.07	2.71	1.24	26.6 (1903) ***	0.61
Online teaching	3.33	0.76	3.65	0.71	19.4 (1903) ***	0.44
Online assessment	3.01	0.95	3.23	1.02	8.51 (1903) ***	0.20
Peer relationship	2.10	0.83	2.26	0.95	7.86 (1903) ***	0.18
Self-efficacy for online learning	3.42	0.82	3.47	0.88	2.81 (1903) **	0.06
Full scale	2.92	0.65	3.18	0.66	19.0 (1903) ***	0.44

M = mean; SD = standard deviation; d = size effect; **p < 0.01; ***p < 0.001.

When performing the comparative analysis between the general expectations of the students (T1) and the experience after the end of the semester (T2), statistically significant differences [*t*(1903) = 19, *p* < 0.001] were found. Hence, students’ experience with ERT at the end of the academic period exceeded their expectations. In this sense, results respond positively to the proposed hypothesis, identifying differences in the scores between T1 and T2.

### Gender Differences in University Students’ Expectations and Experiences of Online Learning During Emergency Remote Teaching

To analyze differences between expectations and experiences considering gender and OECD area, the presence of statistically significant bidirectional interactions was assessed. Subsequently, we performed *post hoc* tests to determine the main effects of gender and OECD area, considering the Bonferroni adjusted *p*-value.

We examined each dimension independently to answer the hypothesis regarding the existence of differences between expectations and experiences related to undergraduate students’ gender during ERT (H_2_). The results showed statistically significant bidirectional interactions among gender and the change in scores between expectations and experiences in the following five dimensions: online learning [*F*(1,1902) = 19.09, *p* < 0.001, GES.002]; comparison with face-to-face education [*F*(1,1902) = 25.23, *p* < 0.001, GES.004]; online teaching [*F*(1,1902) = 5.31, *p* < 0.001, GES.0006]; peer relationship [*F*(1,1902) = 6.79, *p* < 0.01]; and self-efficacy for online learning [*F*(1,1902) = 4.836, *p* < 0.05, GES.0006]. In the case of the online assessment dimension, no statistically significant results were observed.

Regarding the main effect of gender, a significant effect for experience, but not for expectations in the following four dimensions was observed online learning: experience [*F*(1,1902) = 10.64, *p* < 0.01, GES.006]. Online teaching: experience [*F*(1,1902) = 8.54, *p* < 0.01, GES.004]. Peer relationship: experience [*F*(1,1902) = 6.55, *p* < 0.05, GES = 0.003] and Self-efficacy for online learning: experience [*F*(1,1902) = 5.37, *p* < 0.05, GES.003].

On the other hand, in the case of comparison with face-to-face education, the results were significant for expectation [*F*(1,1902) = 13.06, *p* < 0.001, GES.007], but not for experience (*p* = 0.06).

The simple main effect of the differences between expectations and experience were also analyzed, observing statistically significant results for women and men in four of the dimensions: online learning, women [*F*(1,1106) = 203, *p* < 0.001 GES = 0.046] and men [*F*(1,796) = 42.1, *p* < 0.001 GES = 0.011]. In the Comparison with face-to-face education, women [*F*(1,1106) = 589.63, *p* < 0.001 GES = 0.15] and men [*F*(1,796) = 169.09, *p* < 0.001 GES = 0.06]. In the Online teaching, women [*F*(1,1106) = 264, *p* < 0.001 GES = 0.06] and the men [*F*(1,796) = 117, *p* < 0.001 GES = 0.03]. In the peer relationship, women [*F*(1,1106) = 57.5, *p* < 0.001 GES = 0.014] and men [*F*(1,796) = 10.1, *p* < 0.01 GES = 0.003].

In the self-efficacy for online learning dimension, statistically significant results were identified only for women [*F*(1,1106) = 13.4, *p* < 0.001 GES = 0.003]. Even though men and women presented higher scores at T2, women showed the most significant change reflecting a positive experience with online education (see Table 4).

**TABLE 4:** Descriptive data on the expectations and experience of university students considering gender.

	Expectation (T1)				Experience (T2)			
	Women		Men		Women		Men	
Variable	*M*	*SD*	*M*	*SD*	*M*	*SD*	*M*	*SD*
Online learning	3.12	0.85	3.17	0.88	3.48	0.79	3.36	0.89
Comparison with face-to-face education	1.84	1.02	2.02	1.13	2.77	1.22	2.64	1.26
Online teaching	3.34	0.76	3.32	0.75	3.69	0.69	3.59	0.75
Online assessment	3.00	0.96	3.03	0.94	3.23	1.01	3.23	1.04
Peer relationship	2.10	0.81	2.10	0.86	2.31	0.95	2.20	0.94
Self-efficacy for online learning	3.42	0.80	3.41	0.84	3.51	0.83	3.42	0.93
Full scale	2.92	0.64	2.93	0.67	3.23	0.63	3.13	0.68

*M and SD represent mean and standard deviation, respectively.*

**FIGURE 1:**
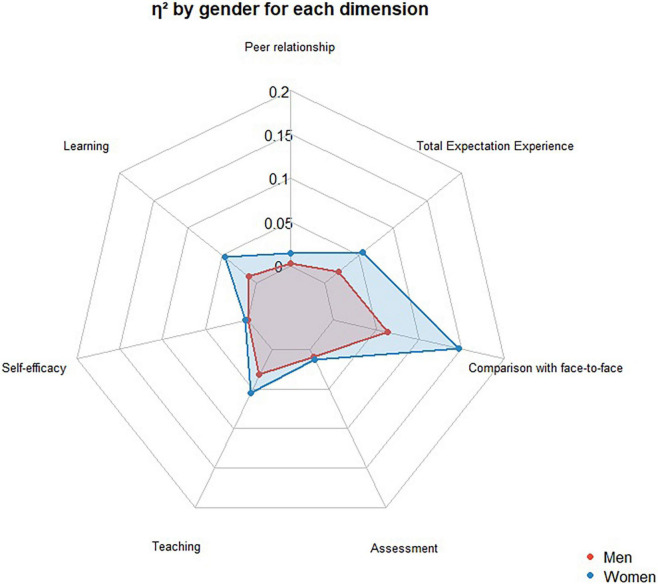
The effect size of the change between expectations and experience according to the gender of the participating student.

Figure 1 shows the size effect identified in the measurements considering gender. In the case of women, we found a large-size effect in the dimension of comparison with face-to-face education and a medium-size effect in the online teaching dimension. In the case of men, the analysis outcomes determine only a medium effect size in the dimension of comparison with face-to-face education and a small size effect in the rest of the dimensions.

### Differences by Disciplinary Area in the Measurement of Undergraduate Students’ Expectations and Experiences of Online Learning During Emergency Remote Teaching

Regarding differences between the scores from expectations and experience of university students during ERT during the COVID-19 pandemic according to disciplinary areas (H_3_), the results by dimension are presented below.

For all six dimensions a statistically significant bidirectional interactions among the OECD area and the differences between T1 and T2 scores was found. The results by dimensions are the following: Comparison to face-to-face education [*F*(5,1898) = 3.54, *p* < 0.01, GES = 0.003], online teaching [*F*(5,1898) = 6.053, *p* < 0.001, GES = 0.004], online assessment [*F*(5,1898) = 7.33, *p* < 0.001, GES = 0.006], online learning [*F*(5,1898) = 8.686, *p* < 0.001, GES = 0.006], peer relationship [*F*(5,1898) = 3.86, *p* < 0.01, GES.003], and self-efficacy for online learning [*F*(5,1898) = 6.99, *p* < 0.001, GES = 0.005].

Regarding the main effect of OECD area, a significant effect for experience and for expectations was observed in the following four dimensions: Comparison to face-to-face education: experience [*F*(5,1898) = 4.43, *p* < 0.01, GES.012] and the expectations [*F*(5,1898) = 9.26, *p* < 0.001, GES.024]: online assessment: experience [*F*(5,1898) = 4.71, *p* < 0.001, GES.012] and expectations [*F*(5,1898) = 3.52, *p* < 0.01, GES.01]; online learning: experience [*F*(5,1898) = 7.4, *p* < 0.001, GES.02] and expectations [*F*(5,1898) = 9.57, *p* < 0.001, GES.03]; self-efficacy for online learning: experience [*F*(5,1898) = 6.22, *p* < 0.001, GES.02] and expectations [*F*(5,1898) = 5.52, *p* < 0.001, GES.01].

Regarding online teaching, a significant effect was observed in expectation [*F*(5,1898) = 4.65, *p* < 0.001, GES.01], but not in experience (*p* = 1). On the other hand, for peer relationship, a significant effect was shown for experience [*F*(5,1898) = 3.67, *p* < 0.01, GES.01] but not for expectations (*p* = 1).

We performed Tukey’s test to assess the differences between OECD areas in expectations and experience. Concerning expectations, the following dimensions presented significant differences (see Table 5). Comparison to face-to-face: Engineering and Technology - Agricultural Sciences *p* < 0.01, Engineering and Technology - Medical and Health Sciences *p* < 0.001, Engineering and Technology - Natural Sciences *p* < 0.05, Engineering and Technology - Social Sciences *p* < 0.001, and Engineering and Technology - Humanities *p* < 0.01. Online teaching: Engineering and Technology - Medical and Health Sciences *p* < 0.01 and Engineering and Technology - Social Sciences *p* < 0.001. Online assessment: Engineering and Technology - Medical and Health Sciences *p* < 0.05. Online learning: Engineering and Technology - Agricultural Sciences *p* < 0.01, Humanities - Medical and Health Sciences *p* < 0.01, Humanities - Natural Sciences *p* < 0.05, Engineering and Technology - Natural Sciences *p* < 0.01, Engineering and Technology - Social Sciences *p* < 0.001, and Engineering and Technology - Humanities *p* < 0.001. Self-efficacy for online learning: Engineering and Technology - Social Sciences *p* < 0.001 and Engineering and Technology - Humanities *p* < 0.01.

**TABLE 5:** Descriptive statistics on students’ expectations and experience during the ERT according to the disciplinary area.

	Agricultural sciences		Medical and health sciences		Natural sciences		Social sciences		Humanities		Engineering and technology	
						
Variable	Expectation	Experience	Expectation	Experience	Expectation	Experience	Expectation	Experience	Expectation	Experience	Expectation	Experience
Peer relationship	2.10 (0.76)	2.12 (0.90)	2.10 (0.80)	2.38 (0.93)	2.10 (0.88)	2.21 (0.93)	2.09 (0.83)	2.30 (0.96)	2.07 (0.93)	1.93 (0.81)	2.11 (0.84)	2.24 (0.99)
Online learning	3.04 (0.93)	3.18 (0.89)	3.18 (0.84)	3.56 (0.82)	3.11 (0.86)	3.35 (0.78)	3.03 (0.88)	3.46 (0.84)	2.74 (0.90)	3.10 (0.76)	3.33 (0.82)	3.45 (0.84)
Self-efficacy for online learning	3.40 (0.81)	3.23 (0.91)	3.47 (0.81)	3.55 (0.85)	3.35 (0.85)	3.40 (0.86)	3.32 (0.81)	3.53 (0.87)	3.17 (0.77)	3.10 (0.84)	3.54 (0.79)	3.50 (0.89)
Comparison with face-to-face education	1.79 (0.99)	2.76 (1.25)	1.89 (1.01)	2.79 (1.18)	1.92 (1.12)	2.63 (1.22)	1.76 (0.99)	2.66 (1.21)	1.61 (0.94)	2.11 (1.03)	2.17 (1.16)	2.83 (1.33)
Online teaching	3.40 (0.80)	3.55 (0.76)	3.27 (0.77)	3.68 (0.75)	3.36 (0.74)	3.64 (0.67)	3.26 (0.78)	3.64 (0.73)	3.23 (0.68)	3.62 (0.60)	3.46 (0.71)	3.67 (0.69)
Online assessment	2.94 (0.99)	2.94 (1.08)	2.92 (0.98)	3.36 (0.97)	3.07 (0.93)	3.19 (1.02)	2.97 (0.94)	3.26 (1.03)	2.81 (0.93)	2.98 (1.02)	3.14 (0.93)	3.21 (1.02)
Total scale	2.91 (0.67)	3.02 (0.69)	2.92 (0.64)	3.27 (0.66)	2.92 (0.65)	3.14 (0.61)	2.85 (0.65)	3.20 (0.65)	2.74 (0.66)	2.92 (0.53)	3.05 (0.64)	3.20 (0.67)

*Results presentation corresponds to mean and standard deviation, in the form Mean (SD).*

In the case of experience, the dimensions that showed significant differences are listed below: Comparison to face-to-face education: Humanities - Agricultural Sciences *p* < 0.01, Humanities - Medical and Health Sciences *p* < 0.01, Humanities - Natural Sciences *p* < 0.05, Humanities - Social Sciences *p* < 0.05, and Engineering and Technology - Humanities *p* < 0.001. Online assessment: Medical and Health Sciences - Agricultural Sciences *p* < 0.001 and Social Sciences - Agricultural Sciences *p* < 0.05. Online learning: Medical and Health Sciences - Agricultural Sciences *p* < 0.001, Social Sciences - Agricultural Sciences *p* < 0.01, Engineering and Technology - Agricultural Sciences *p* < 0.01, Natural Sciences - Medical and Health Sciences *p* < 0.05 Humanities - Medical and Health Sciences *p* < 0.001, Humanities - Social Sciences *p* < 0.05, and Engineering and Technology - Humanities *p* < 0.05. Peer relationship: Humanities - Medical and Health Sciences *p* < 0.01 and Humanities - Social Sciences *p* < 0.05. Self-efficacy for online learning: Medical and Health Sciences - Agricultural Sciences *p* < 0.01, Social Sciences - Agricultural Sciences *p* < 0.01, Engineering and Technology - Agricultural Sciences *p* < 0.05, Humanities - Medical and Health Sciences *p* < 0.01, Humanities - Social Sciences *p* < 0.01, and Engineering and Technology - Humanities *p* < 0.01.

Finally, the simple main effect of the differences between expectations and experience for each dimension was analyzed, observing in some cases statistically significant effects for all six OECD areas, while in others only for one (see Table 5). The results reflected by the analysis are listed by dimension: Comparison to face-to-face education: Agricultural Sciences [*F*(1,140) = 71.71, *p* < 0.001, GES = 0.16], Medical and Health Sciences [*F*(1,415) = 227.33, *p* < 0.001, GES = 0.14], Natural Sciences [*F*(1,311) = 93.81, *p* < 0.001, GES.08], Social Sciences [*F*(1,508) = 247.639, *p* < 0.001, GES = 0.14], Humanities [*F*(1,60) = 11.93, *p* < 0.01, GES = 0.06], and Engineering and Technology [*F*(1,464) = 97.77, *p* < 0.001, GES = 0.06]. Online teaching: Agricultural Sciences [*F*(1,140) = 8.14, *p* < 0.05, GES = 0.01], Medical and Health Sciences [*F*(1,415) = 126, *p* < 0.001, GES = 0.07], Natural Sciences [*F*(1,311) = 58.2, *p* < 0.001, GES.04], Social Sciences [*F*(1,508) = 124, *p* < 0.001, GES = 0.06], Humanities [*F*(1,60) = 23.8, *p* < 0.001, GES = 0.09], and Engineering and Technology [*F*(1,464) = 51.6, *p* < 0.001, GES = 0.02].

The following differences in the dimension of online assessment between discipline areas were found: Medical and Health Sciences [*F*(1,415) = 70.57, *p* < 0.001, GES = 0.05] and Social Sciences [*F*(1,508) = 37.89, *p* < 0.001, GES = 0.02]. Online learning: Medical and Health Sciences [*F*(1,415) = 86.1, *p* < 0.001, GES = 0.05], Natural Sciences [*F*(1,311) = 26.4, *p* < 0.001, GES.02], Social Sciences [*F*(1,508) = 131, *p* < 0.001, GES = 0.06], Humanities [*F*(1,60) = 9.94, *p* < 0.05, GES = 0.5], and Engineering and Technology [*F*(1,464) = 10.8, *p* < 0.01, GES = 0.006]. Peer relationship: Medical and Health Sciences [*F*(1,415) = 42.1, *p* < 0.001, GES = 0.024], Social Sciences [*F*(1,508) = 27, *p* < 0.001, GES = 0.014], and Engineering and Technology [*F*(1,464) = 9.88, *p* < 0.05, GES = 0.005]. Self-efficacy for online learning: Social Sciences [*F*(1,508) = 29.4, *p* < 0.001, GES = 0.02].

**FIGURE 2:**
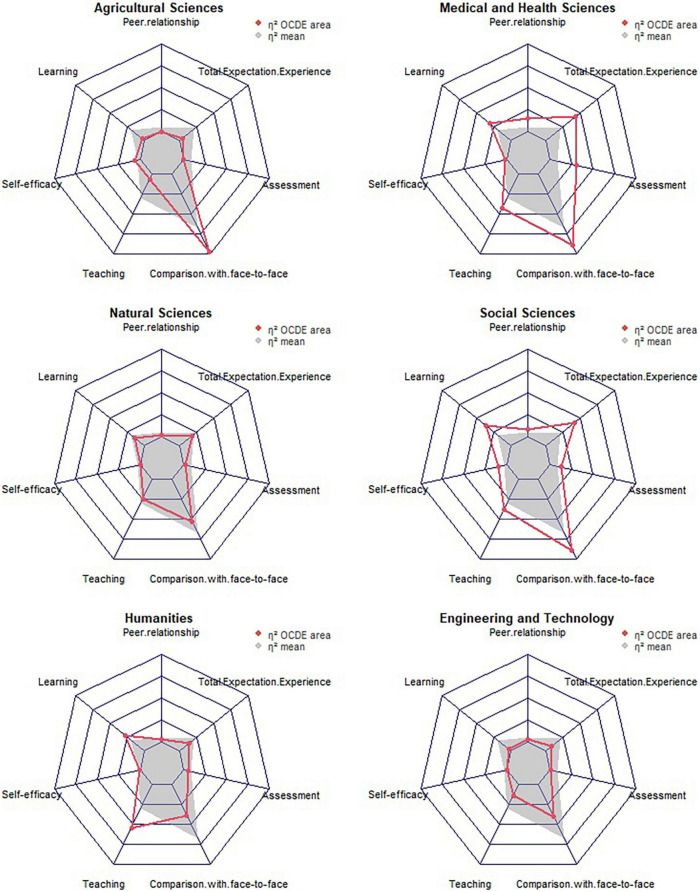
Size effect regarding the change between expectations and experience according to the disciplinary area.

Figure 2 shows the size effect identified considering the OECD area. In Agricultural Sciences, we found a large-size effect in the dimension of comparison with face-to-face education and a small effect size in the dimensions of online learning, self-efficacy for online learning, online teaching, and the full scale. There were no effects detected in the rest of the dimensions. In Medical and Health Sciences, the analysis outcomes reflected a large-size effect in comparison with face-to-face education and a medium-size effect in the dimensions of online teaching and full scale. In addition, we found a small effect in the dimensions of peer relationship, online learning, and online assessment. In Natural Sciences, we found a medium-size effect in the size of comparison with face-to-face education and a small-size effect in online teaching, online learning, and full scale. No effects on the remaining dimensions were found. In the case of Social Sciences, we found a large-size effect for comparison with face-to-face education, a medium-size effect in the dimensions of online learning, online teaching, and the full scale, and a small-size effect in the rest of the dimensions. The Humanities area presented a medium-size effect in online teaching and comparison with face-to-face education dimensions and a small-size effect in online learning, peer relationship, online evaluation, and full scale. Finally, in Engineering and Technology, a medium-size effect in the dimension of comparison with face-to-face education and a small-size effect in the online teaching, online learning, peer relationship dimensions, and full scale were identified. In the rest of the dimensions, there were no effects detected.

## Discussion

Due to the COVID-19 pandemic, the transition to ERT impacted students’ expectations and experiences during their professional training. This research aimed to analyze students’ expectations and experiences considering the gender and disciplinary area of the participants. Findings are analyzed and discussed in terms of the hypotheses raised in section “The Present Study.”

### Differences Among University Students’ Expectations and Experiences During the Emergency Remote Teaching Produced by the COVID-19 Pandemic

Changes between students’ expectations and experiences during ERT were found. Students’ expectations at T1 about online education were negative. However, at the end of the academic period, students indicated having a positive experience in most studied dimensions. They only showed a negative experience regarding the relationship with their peers and the comparison with face-to-face.

Several studies during the pandemic point out the lack of confidence toward the different educational actors and online education opportunities. This mistrust is associated with a lack of knowledge of the modality and its advantages (Villa et al., 2020) and little awareness of the available virtual educational tools (Rahiem, 2020; Almomani et al., 2021). In addition, the unexpectedness of the transition was a challenge for teachers and students, generating a problematic, improvised, and intuitive confrontation (Barbour et al., 2020; Hodges et al., 2020).

Students’ perception of the limited opportunities virtual classrooms and other technological tools provided them to interact and work collaboratively with peers is particularly noteworthy. Several reports emphasize the benefits of cooperative work versus a competitive or individualistic methodology in higher education. The former generates better learning and significant commitment and involvement in academic tasks (León del Barco et al., 2017; Guerra Santana et al., 2019; Hamdan et al., 2021). Also, collaborative work is closely related to desired competencies in the profession’s exercise, an aspect that is not present in this study. In this context, the literature describes technological mediation in education to provide significant possibilities of simultaneous sociability, of connection between communities and people, subscription, and asynchronous communication that generates network effects that tend to accelerate individuals and group learning (De Haro, 2010; Anthony et al., 2019). Therefore, it is crucial to understand why peer interaction during ERT was negatively perceived, especially considering that the LMS had the functionalities for such activities. We believe that it is partly a product of the little knowledge of these tools by both teachers and students.

The observation that students face online education with a high sense of self-efficacy, believing that they have the skills to respond to the learning challenges that this modality presents, could be explained by the lack of knowledge and experience, as well as underestimating the necessary skills. Consequently, students perceive a lower complexity than the real one, as described by the “Durning Kruger effect” (Dunning, 2011). It is possible that by the regular use of technology, social media, phones, and computers, they initially self-perceived as more competent.

The perception of a better experience concerning the initial expectation suggests that the implementation of ERT, although not devoid of difficulties, responded to students’ needs. Hence, higher education institutions’ response and the teachers’ and students’ adaptation adequately provided a well-perceived learning environment. Furthermore, the above is consistent with other research during the pandemic that reported positive experiences by teachers and students in terms of having been able to face the educational process despite the adversities of the confinement and its urgency (Sepulveda-Escobar and Morrison, 2020).

We can conclude that the educational community and higher education authorities have learned greatly during ERT. Therefore, it will be interesting to study how to translate these lessons into explicit guidelines and practices when returning to normality post-pandemic.

### Gender Differences in University Students’ Expectations and Experiences of Online Learning During Emergency Remote Teaching

When evaluating changes in expectation and experience scores considering the sex of the participants, at the beginning of ERT, men and women presented similar levels of expectations about online education. However, experiences showed differences according to gender. Although both perceived the educational experience as positive, women gave higher values than men, in the dimension with lower punctuation in the experience compared with a face-to-face modality and peer relationship.

These results are consistent with the study reported by Almomani et al. (2021), conducted during the COVID-19 pandemic, and reports that women students were more optimistic, satisfied, and committed to the online learning experience than men students during this period. Furthermore, a 62-country study on the impact of the pandemic on higher education (Aristovnik et al., 2020) reports a minor negative impact of confinement on women students’ learning, adaptation, and relationship with the teachers. In this study, a similar result was obtained regarding the perception of online teaching. Women students presented a higher value of the teacher’s commitment to ERT. Women considered that instructors were available and attentive to their learning needs, complied with the course syllabus, and made good use of the available virtual classroom tools.

In another study on online university education in the context of COVID-19 (Shahzad et al., 2021), the authors were able to identify differences between men and women regarding the perception of usefulness, ease, and satisfaction with the use of the learning management systems provided by the institution. This finding suggests that adaptation processes to university life in electronic learning environments may be different for men and women. Therefore, this information could be valuable for university authorities to strengthen and improve the university system support.

### Differences in Students’ Expectations and Experiences by Disciplinary Area of Online Learning During Emergency Remote Teaching

Research on the effects of the COVID-19 pandemic in the context of higher education has identified significant challenges for implementing online education, such as inequality, funding, and ways to develop learning in general (Aristovnik et al., 2020; Funk, 2021). In this context, it is essential to identify if these challenges and opportunities are specific to a particular disciplinary area or apply to the general community. Thus, differences during ERT between disciplinary areas were analyzed.

Differences in the expectations and experiences of university students in the six disciplinary areas classified according to their undergraduate programs were found. Unfortunately, there is little literature on the influence of the disciplinary area to which students’ undergraduate programs belong regarding experience with online education in ERT. Knowing about students’ experience in each disciplinary area will allow teachers and educational authorities to identify weaknesses and good practices that will otherwise not be detected to design and develop monitoring plans and improve the quality of online education in the future.

We found differences within expectations in the online teaching dimension for all disciplinary areas. On the other hand, Students from Engineering and Technology and Medical and Health Sciences areas reported higher experience scores in this dimension, which implies that these students felt more confident about the actions performed by their instructors. This result could be related to the use of technology by Engineering and Technology teachers and the teacher training in the medical education area, often advanced.

Despite the improvement between student expectations and experiences of the online assessment dimension, changes presented null (Agricultural Sciences, Natural Sciences, and Engineering and Technology) or small (Social Sciences, Humanities, and Medical and Health Sciences) size effect. The assessment processes continue to be an area of concern. Other reports support this statement. For example, Jordanian university students perceived that assessment during the pandemic allowed them to obtain higher grades than face-to-face assessments. Nonetheless, most students perceived that the evaluative processes were unfair and learned more minor than the quality reflected (Almomani et al., 2021). Consistently, a study conducted with 8265 Chilean university students (Lobos et al., 2022) reported that students perceived a bad experience regarding the assessment process during the pandemic. Again, researchers observed a greater expectation of obtaining a good grade rather than of achieving learning. As a result, students considered that they failed to achieve good quality training. Despite these findings, a study carried out in Chile indicates that students’ academic performance improved compared to the previous academic period (Franco et al., 2021). Therefore, the guidelines and strategies used by teachers regarding assessment continue to be an essential element to consider in the design of quality online education.

An interesting finding is a large-size effect obtained in the differences between the scores of expectations and experience of students of Agricultural Sciences and Medical and Health Sciences, for the comparison with face-to-face education dimension. Further research is required to identify good practices teachers and students implement in undergraduate programs classified in these two OCDE discipline areas.

We believe that the differences in the results of the students’ expectations and experience according to the disciplinary area are due to the different challenges encountered in the adaptation of the courses (efficient ones). Accordingly, strategies used, for example, in Health Sciences, can be used in realistic training scenarios that relate to people (Social Sciences and Humanities). One of these strategies can be using remote standardized patients who have meetings with students through the Internet. These activities allow teachers and standardized students to have spaces for evaluation and feedback (Langenau et al., 2014; Bączek et al., 2021). This technique could be adapted to other teaching contexts using work situations in the training of other professionals.

Concerning the dimension of self-efficacy for online learning, no significant changes in four of the six knowledge evaluated areas were observed. Agricultural Sciences and Social Sciences displayed differences with small-size effect. Thus, ERT did not increase students’ confidence beliefs toward taking classes in the online teaching modality.

Despite valuable information that has been obtained for this study, some limitations are identified. First, the results presented correspond to university students’ responses from a single educational institution, so the interventions of university authorities could bias expectations and subsequent experiences in the context of ERT. Second, it was not part of this study to evaluate access gaps and other student variables that could affect the results. Finally, variables associated with the teacher or course characteristics that may influence the outcomes could not be controlled. Therefore, the results aim to study changes between students’ expectations and experience in an exploratory way. Other studies must consider the assessment of student (e.g., difficulties in accessing online classes), professor (e.g., profession), or course (e.g., type, time commitment) variables that may affect undergraduate expectations and experiences.

### Study Implications

In this research, we found that students’ experiences with online education during the ERT were more optimistic than their expectations at the beginning of the semester. For this reason, the results found, together with other sources of institutional information such as learning analytics and institutional indicators, will allow authorities and teachers to develop guidelines to promote quality online education. It is also possible that university authorities could consider these preferences to design and create online courses for their students (Zapata-Cuervo et al., 2021).

The relationship with peers and professors is still considered a weak point of online education. This is a crucial aspect to be addressed by university professors. In the context of virtuality, professors need to maintain communication channels that allow them to provide students with timely feedback from online video tutorials or email guides after class (Bao, 2020; Vladova et al., 2021b). We identified statistically significant differences in the experiences of men and women. This represents an opportunity to investigate how the characteristics of each student improve academic performance and decrease the probability of dropping out of college.

We found differences in the students’ experiences according to the scientific areas. These results translate into a challenge to identify the strategies and actions that facilitated a positive experience to replicate them in similar formative contexts. Further, studies can be performed to identify good practices applied in general contexts and those appropriate for each discipline. Higher education institutions are expected to accompany teachers and students in the different scientific areas during the post-pandemic academic continuity. Exceptional support is scheduled in aspects such as planning and prioritization of practical classes, promoting a combined approach of virtual and face-to-face education (Pham and Ho, 2020; Vladova et al., 2021b).

Future research could assess how students’ variables (e.g., internet access, type of device used to study), courses’ factors (e.g., number of hours of dedication, learning goals, instructional design, type of materials, or shared resources), teachers’ aspects (e.g., technological acceptance, use of strategies, training) or the institution’s elements (e.g., promotion of teaching through technology, support for students and teachers, use of online learning platforms, technological campuses) impact the expectations and subsequent experience of students during the development of online courses., especially regarding strength and weaknesses according to discipline areas.

The findings of this work contribute to identifying dimensions and areas that require special attention to establish preventive and corrective actions by university authorities for the near future and propose the opportunity of further studying good practices of better-perceived experiences of discipline areas.

## Conclusion

The students’ experiences during ERT due to the COVID-19 pandemic exceeded expectations. Students reported high expectations about their self-efficacy to cope with this new scenario, even though low expectations regarding peer relationships, online teaching, and comparison with face-to-face education were observed concerning the experience after the semester. Students indicated positive experiences with online learning and teaching. They felt that the professor provided adequate support in terms of education, instruction, and assessment. Negative experiences persisted regarding peer relationships and the overall experience compared to face-to-face teaching. Additionally, men and women presented similar expectations at the beginning of the semester regardless of their discipline, while women were more optimistic about educational experiences during ERT. Finally, concerning the disciplinary area, differences in most of the assessed dimensions were observed, representing an opportunity to study further and identify good practices in those dimensions and disciplines that presented positive perception and effect.

## Data Availability Statement

The original contributions presented in the study are included in the article/supplementary material, further inquiries can be directed to the corresponding author.

## Ethics Statement

The studies involving human participants were reviewed and approved by Institutional Ethics Committee of University of Concepción. The patients/participants provided their written informed consent to participate in this study.

## Author Contributions

KL and RC-R: conceptualization. KL, RC-R, and CB: methodology. JM-N: formal analysis and visualization. KL, RC-R, and AM-T: research and writing—preparing the original draft. AM-T, CB, and CF: resources, project management, and fundraising. JM-N and RC-R: data curation. CB and CF: writing—revising and editing. KL, CB, and CF: monitoring.

## Conflict of Interest

The authors declare that the research was conducted in the absence of any commercial or financial relationships that could be construed as a potential conflict of interest.

## Publisher’s Note

All claims expressed in this article are solely those of the authors and do not necessarily represent those of their affiliated organizations, or those of the publisher, the editors and the reviewers. Any product that may be evaluated in this article, or claim that may be made by its manufacturer, is not guaranteed or endorsed by the publisher.

## Footnotes

1 Lobos, K., Cobo-Rendon, R., Cisternas, N., Aslan, J., and López Angulo, Y. (under review). *Experiences With Online Education of College Students During Emergency Remote Education Due to COVID-19*.
